# Perceptions, knowledge, and communication preferences about indoor mold and its health implications among persons affected by Hurricane Harvey: a focus group analysis

**DOI:** 10.1186/s12889-022-13603-0

**Published:** 2022-06-15

**Authors:** Pooja Gandhi, LaQuita Malone, Samantha Williams, Callie Hall, Kirstin Short, Kaitlin Benedict, Mitsuru Toda

**Affiliations:** 1 ASRT, Inc., assigned to Mycotic Diseases Branch, Division of Foodborne, Centers for Disease Control and Prevention, Waterborne, and Environmental Diseases, National Center for Emerging and Zoonotic Infectious Diseases, 1600 Clifton Road NE, Mailstop H24-9, Atlanta, GA 30329 USA; 2 Houston Health Department, 901 Bagby Street, Houston, TX 77002 USA; 3 Mycotic Diseases Branch, Division of Foodborne, Waterborne, and Environmental Diseases, National Center for Emerging and Zoonotic Infectious Diseases, Centers for Disease Control and Prevention, 1600 Clifton Road, Atlanta, GA 30329 USA

**Keywords:** Disasters, Mycoses, Focus Groups, Qualitative Research, Health Communication

## Abstract

**Background:**

Among people affected by Hurricane Harvey, we assessed experiences and perceptions (e.g., knowledge, attitudes, and practices) regarding mold and its impact on health and elicited participants’ opinions about how to improve public health messaging about indoor mold after a large flooding event.

**Methods:**

Houston Health Department conducted four focus groups with 31 Houston metropolitan area residents during January to March 2020, using a semi-structured discussion guide and federal communication materials about indoor mold. Drawing from a theoretical framework analysis, transcripts were grouped into relevant themes using inductive and deductive coding.

**Results:**

Hurricane Harvey had a large impact on participants’ living standards, and widespread financial barriers to remediation led to long-term mold exposure for many participants. Knowledge about mold’s impact on health and proper mold clean-up practices varied, and clean-up behaviors did not commonly align with federal guidance. Participants generally preferred traditional forms of outreach, such as in-person, radio, and television announcements, to communicate public health messaging.

**Conclusions:**

More strategic dissemination of expanded public health educational materials about proper mold clean-up practices and the health risks of mold exposure following flooding events is needed.

## Background

In August 2017, Hurricane Harvey destroyed over 200,000 homes and businesses throughout the Houston metropolitan area, causing $125 billion in damage and displacing over 30,000 people [1–4]. Exposure to indoor mold is a serious concern after hurricane-related flooding [5]. Mold can exacerbate allergies or asthma and cause serious invasive mold infections (IMIs), which cause high morbidity and mortality in immunocompromised people [5]. People at highest risk for IMIs include those who have recently undergone organ or stem cell transplants or major surgery, people with other immunocompromising conditions such as neutropenia, and people with certain other pre-existing conditions such as uncontrolled diabetes [6–11].

Federal agencies, including the Centers for Disease Control and Prevention (CDC), recommend that immunocompromised people avoid areas and buildings with indoor mold after disasters [6, 12]. Despite this recommendation, one study showed that half of surveyed immunocompromised people cleaned up indoor mold after Hurricane Harvey. Most of them did not wear respiratory protection, despite hearing or reading messages about what to wear while cleaning up mold [5]. In the same study, common sources of these messages included television, word of mouth, and health care providers, but social media and websites were not mentioned [5]. In contrast, other studies showed that social media was a common method for sharing information during Hurricane Harvey, though preferences can vary by demographic features, such as age [13–16].

Many interrelated factors, including socioeconomic status (SES), perceived health risks, knowledge, access to personal protective equipment (PPE), and perceived benefits of indoor mold clean-up likely affect adherence to federal recommendations about how to clean up mold safely [5, 17]. Some circumstances may prevent any mold remediation in a timely manner or the ability to leave the home during the process. To better understand these factors, we conducted focus group discussions (FGDs) with people affected by Hurricane Harvey to assess their perceptions and short-term and long-term experiences regarding indoor mold and its health effects as well as perceptions of professional mold testing, which is not recommended by CDC or the Environmental Protection Agency (EPA). FGDs also aimed to elicit opinions about preferred methods of communication and public health messaging about indoor mold.

## Methods

The Houston Health Department (HHD) recruited participants through its website and social media accounts. Participants were at least 18 years of age and resided in Houston during Hurricane Harvey. HHD also leveraged the assistance of community partners and its internal Area Agency on Aging, which has a large community network. One of the partners was the Harris County Long-Term Recovery Committee, composed of local government organizations (e.g., City of Houston) and faith-based and non-profit organizations. Two HHD staff members facilitated each of four FGDs during January through March 2020. Each FGD was audio-recorded. Participants provided written and verbal informed consent. No incentives were given. This activity was reviewed by CDC and was conducted in accordance with applicable federal law and CDC policy.

### Data collection

A semi-structured discussion guide and federal communication materials about mold clean-up were used during the FGDs. Participants were asked a series of open-ended questions about their experiences related to hurricane-related damages, what information would have been useful following the flooding, and how local health departments could best communicate that information. Participants were also asked to provide feedback on messaging about mold in a post-hurricane setting. Three FGDs were conducted in English and one in Spanish. Data were transcribed verbatim, and Spanish FGDs were transcribed and translated by bilingual speakers.

### Data analysis

Using an approach informed by Grounded Theory [18], data were coded through an integrated deductive and inductive coding process and were analyzed using Nvivo12 (QSR International, United States) [19]. Pre-determined codes were developed based on the topics covered in the FGD guide, and additional codes were developed after transcript review. These codes were further stratified into more specific sub-codes and also combined into more general nodes. A comprehensive list of nodes, codes, and sub-codes was developed through team consensus, and themes were developed and used to group similar codes and sub-codes that were prominently featured during FGDs (Table 1).

**Table 1:** Focus group codebook

Themes/Subthemes	Nodes	Codes	Sub-codes
**Theme 1**	**Impact of Hurricane Harvey on standards of living**	Financial issues with remediation of damage from Harvey	Currently conducting remediation
**Subtheme 1**		Frustration with lack of local assistance	
**Theme 2**	**Mold’s impact on health**	Perceived severity of mold-related health issues	
		Perceived susceptibility to mold-related health issues	
**Theme 3**	**Use of personal protective equipment (PPE)**	Wearing long sleeves	Wearing long sleeves as an actionable behavior
		Use of masks	Lack of use of masks
**Subtheme 2**	**Professional testing for mold**	Conducted professional testing	
		Did not conduct professional testing	
**Theme 4**	**Messaging about mold**	Preferred methods of receiving mold messages	Radio
			Television
			Telephone
			Word of mouth
		Timing of messages	

Themes were developed from corresponding nodes, subthemes were developed from corresponding codes

### Theoretical framework

**Fig. 1:**
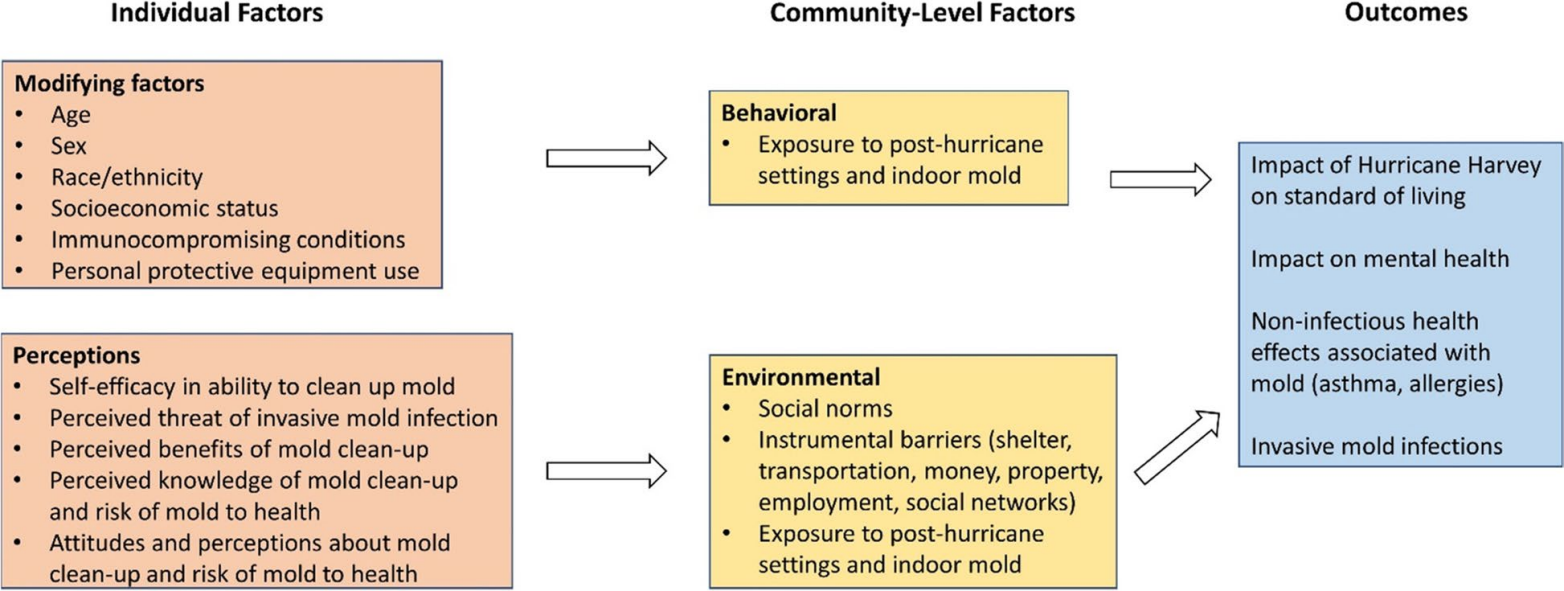
Factors influencing risk of invasive mold infection

The semi-structured discussion guide was developed using an overarching theoretical framework describing factors contributing to risk of IMI (Fig. 1). The framework was developed based on guiding principles and considerations from the Health Belief Model (HBM), [20, 21] the Extended Parallel Process Model (EPPM), [20, 22] and the PRECEDE-PROCEED Model (PPM) [20, 23]. The framework consists of three major components: individual factors, community-level factors, and health outcomes. Components of this framework pulled from the HBM include self-efficacy (in ability to clean up mold); perceived threat (of IMI); and perceived benefits (of mold clean-up and risk of mold to health) [24]. Similarly, perceived severity and susceptibility and self-efficacy are key to the EPPM [22]. The PPM highlights factors related to quality of life, behavioral and environmental factors, pre-disposing factors (e.g., self-efficacy, attitudes, beliefs), which informed the overall structure and components selected for this framework [23].

## Results

Thirty-one Houston residents participated in the four FGDs. Six (19%) were men, 19 (61%) were women, and 6 (19%) did not report their gender. Most participants identified as Black or African American (48%) or Hispanic or Latino (29%). The majority of participants were more than 60 years old (58%) and were homeowners (70%) at the time of Hurricane Harvey. More than half of the participants had no education beyond high school (55%) (Table 2).

**Table 2:** Demographic features of focus group participants

*n* = 31	
**Sex**	
Male	6 (19%)
Female	19 (61%)
No response	6 (19%)
**Race/Ethnicity**	
Black or African American	15 (48%)
Hispanic or Latino	9 (29%)
White	1 (3.2%)
Unknown	3 (10%)
Multiple races/ethnicities	3 (10%)
**Age**	
30–49 years	1 (3%)
50–59 years	3 (10%)
60–79 years	14 (45%)
> 80 years	4 (13%)
No response	9 (29%)
**Highest Level of Education Completed**	
No high school	3 (10%)
Some high school	3 (10%)
High school graduate/GED	11 (35%)
Technical school/certificate	0
Some college/associate degree	4 (12%)
College graduate/bachelor’s degree	5 (16%)
Post-graduate/professional degree	2 (6%)
No response	3 (10%)
**Home Ownership at the time of Hurricane Harvey**	
Rent	6 (19%)
Own	22 (71%)
Other/no response	3 (10%)

Four dominant themes and two subthemes emerged from FGDs (1) Hurricane Harvey’s impact on standards of living, (2) mold’s impact on health, (3) knowledge and practices surrounding PPE use when cleaning up mold, and (4) preferred methods for receiving messages about mold. Subthemes included (1) financial issues associated with remediating mold and (2) lack of professional mold testing. The themes and subthemes demonstrated a range of individual and community-level factors that contributed to the participants’ risk of IMI as illustrated in Fig. 1. Individual factors included modifying factors such as (1) socioeconomic status (Theme 1, Subtheme 1), (2) PPE use (Theme 3); and perceptions such as (1) perceived threat of IMI (Theme 2), (2) perceived benefit of mold clean-up (Theme 2, Theme 3), (3) perceived knowledge of mold clean-up and risk of mold to health (Theme 2, Theme 3), and (4) attitudes and perceptions about mold clean-up and risk of mold to health (Theme 2, Theme 3). Community-level factors included behavioral factors such as (1) exposure to post-hurricane settings and indoor mold (Theme 1); and environmental factors such as (1) social norms (Theme 4) and (2) instrumental barriers (Theme 1, Subtheme 1). Key representative quotes were chosen to illustrate each of these themes and subthemes.

### Theme 1: Impact of Hurricane Harvey on standards of living

Participants described experiences with unrepaired damage to their homes, ongoing remediations, financial issues, and a need for increased local assistance three years after Hurricane Harvey.

“We are not even in recovery stage because we are still affected from Harvey. [Many] houses from the affected [areas still] need to be torn down.”

—Participant A, Focus Group #1

“If [the] mold [still hasn’t been remediated] since 2017, it’s not going anywhere.”

—Participant B, Focus Group #2

### Subtheme 1: Financial issues associated with remediating mold

Participants described financial concerns, primarily lack of insurance or inability to afford mold remediation.

"I’m disabled and on limited income. I have insurance and other bills [to pay], so I have no choice [but to live in my house with mold since] no [other] funding available.”

—Participant B, Focus Group #1

“Yes, had to go into pocket [because] insurance would not cover. Had to buy bleach, apple cider vinegar, [and] lemon juice.”

—Participant A, Focus Group #4

“I don’t have insurance the house is on blocks and the insurance is expensive. They ask about my check, but the check is for the whole month. I can’t do everything.”

—Participant B, Focus Group #3

### Theme 2: Mold’s impact on health

Participants expressed a perceived knowledge about how mold can impact health, and many seemed to understand that mold can be harmful to health.

“[Mold is] harmful to health so you have to be protected.”

—Participant D, Focus Group #1

However, inaccurate statements regarding the inability to recover from a mold-related illness and the deadly outcomes of certain types of mold demonstrated that the knowledge about mold’s effect on health was limited.

“There’s no recovery. We won’t know how it affects us until years from now.”

—Participant B, Focus Group #4

“If you have black mold in your house it’s killing you and [you] have to throw everything away”

—Participant C, Focus Group #1

### Theme 3: Knowledge and practices surrounding PPE use when cleaning up mold

FGD results highlighted conflicting knowledge and practices regarding PPE use. Participants described using certain PPE when cleaning up mold, primarily masks, though some stated that masks were not necessary, or that an alternative form of PPE, such as long sleeves, would suffice.

"The mask is not necessary but need to cover the hands. We used a handkerchief or bandana, not a mask."

—Participant C, Focus Group #3

"We didn't use masks when we cleaned."

—Participant B, Focus Group #3

“[A] mask may do for a little while, [but] you can pass out or clog the mask up. Masks are [actionable] because may need a different type. I breath from my mouth, not my nose.”

—Participant C, Focus Group #4

### Subtheme 2: Lack of professional testing of mold

CDC and other federal agencies do not recommend professional testing for mold in most cases [12]. Although a few participants had their home professionally tested for mold, most participants did not. However, some participants who did not have their homes professionally tested cited financial issues as the reason, rather than the CDC guidance.

“[A] company, they did some type of test, said I had mold.”

—Participant E, Focus Group #4

“No, I did not get testing. It’s too expensive.”

—Participant D, Focus Group #3

“[Participant E] was blessed. I priced it out, and [decided to] use bleach.”

—Participant B, Focus Group #4

“All of us have mold. Can you refer us to a company to inspect for mold? [The] city [should pay] or give us a discount.”

—Participant C, Focus Group #2

### Theme 4: Preferred methods of receiving messages about mold

Preferred methods of receiving messages about mold included in-person outreach, radio and television announcements, printed flyers, phone calls, and emails. Participants’ suggestions for printed messages included flyers distributed at grocery stores and local community centers, and with mailed bills. Some participants also expressed interest in messaging about hurricane recovery and how to get local assistance in general specifically targeted to elderly populations.

“TV or radio. Not all of us have internet.”

—Participant C, Focus Group #3

"People on the ground in the neighborhood. Knock[ing] on the doors, alerting the community."

—Participant D, Focus Group #4

Participants also commented that existing CDC and local public health messaging did not address how to deal with long-term mold exposure and that the intended target audience for the messaging was unclear.

## Discussion

The FGDs, involving predominantly older Black and Hispanic Houston residents who had experienced long-term mold exposure, provided insight into participants’ perceptions about indoor mold, its health impacts, mold clean-up, and communication preferences after Hurricane Harvey. FGDs highlighted large impact on participants’ living standards. Mold was a widespread issue, and financial limitations were a prominent barrier to mold remediation and rebuilding in general. Participants generally expressed a preference for more traditional methods of communication during emergencies over more contemporary methods such as Internet and social media. Comments also pointed to a gap in existing communication materials to address people who live with long-term mold exposure.

Emergent themes and subthemes reflected participants’ experiences with several individual and community-level factors contributing to risk of IMI (Fig. 1). Although participants expressed perceived knowledge of mold’s health risks and a perceived threat of mold to one’s health, instrumental barriers such as access to financial and material resources may have restricted their ability to take certain prevention measures. Participants’ knowledge of recommended mold clean-up practices and their likelihood to follow federal guidance may have been influenced by the way mold clean-up messaging was disseminated. The lasting effects of Hurricane Harvey on participants’ living situations nearly three years after the hurricane were widely apparent, as participants described unrepaired damage or living in their homes during ongoing repairs. Previous research similarly found that one year after the hurricane, most people (~ 65%) still had unrepaired damage [25]. In this study, financial barriers were commonly mentioned as a factor impacting mold clean-up and general repairs. As such, participants reported that federal guidance to avoid mold was not actionable without the ability to access to financial assistance.

Although substantial financial aid opportunities were available from federal, state, and local agencies, as well as non-profit and international organizations, Houston residents faced challenges in navigating these systems. Technical difficulties compounded the procedural issues in the immediate aftermath of the flooding, as many people lacked reliable phone service or Internet access for several weeks to months following Hurricane Harvey. The struggle to access available funds persisted months to years after the storm. Thus, despite resource availability, many residents had yet to receive local or state assistance for home damage repairs at the time of the FGDs, leading some to feel overwhelmed and discouraged. Practical guidance from local and city authorities on how to effectively acquire financial aid may help expedite recovery for future events.

CDC and EPA recommend that all indoor mold growth should be cleaned up appropriately given its potential to cause health problems and that persons should wear PPE while cleaning up mold including fit-tested N95 respirators if possible [12]; immunocompromised people, who are at risk for severe infections if exposed to mold, should avoid contaminated buildings as much as possible.^12^ Some participants seemed to believe that using PPE when remediating mold was important. However, discrepancies arose between knowledge and actions; some participants also described the importance of wearing masks when remediating mold but did not claim to practice the actual behavior. Notably, the FGDs were conducted prior to the COVID-19 pandemic, and mask perception and use may differ in a post-pandemic setting. Discussion questions did not distinguish between different types of masks. The inconsistency between knowledge about the benefits of wearing PPE when remediating mold and participants’ actions could be a result of many factors, including instrumental barriers as well as low perceived threat of IMI and non-IMI health effects associated with mold, such as asthma or allergies (Fig. 1). These findings aligned with prior research that most participants who cleaned up mold after Hurricane Harvey did not wear respiratory protection (i.e., masks), but reported gloves as their most frequently used form of PPE [5].

Although CDC and the EPA do not recommend professional mold testing, FGD participants expressed concern that they could not afford to have their homes tested for mold. Accordingly, most participants did not have mold testing done. Most participants were older (ages > 60), of minority status, and had no education beyond high school, which may have impacted accessibility to mold testing. A previous study showed that almost half (~ 47%) of people affected by Hurricane Harvey had professional mold testing in their home [25].

Participants’ preferred methods of communication about mold, its health effects, and proper clean-up included mostly traditional modes of outreach such as in-person, radio, and television announcements; printed flyers; and phone calls, though emails were also noted as a favored communication tool. This finding seems to contrast with prior reports that social media was widely used after Hurricane Harvey, and increasingly, after other natural disasters, to share information and request help [26–28]. Communication preferences have been shown to vary substantially across demographic characteristics, and our results are likely explained by the older demographic makeup (> 60 years) of FGDs. Older adults tend to have lower social media penetration rates and often prefer television as their main media resource for information, followed by radio and Internet, although a previous study showed 58% of older adults (> 70 years) were not familiar with their state’s official disaster website [29, 30]. Younger adults (18–34 years) are least 3 times more likely than those aged 35–65 years old to report using the Internet as their primary source of information [29]. Internet access, in general, also tends to be higher in persons with higher SES; persons with lower education levels tend to prefer more traditional modes of outreach, while those with higher education levels tend to prefer Internet sources as their primary source of information [29]. Other demographic features like sex and race and ethnicity can also affect information-seeking behavior. For example, females are more likely to discover information on social media and share information via email, text, or other online methods, while Hispanics or Latinos tend to prefer radio as a means of information [29, 31]. Given the wide range of communication preferences, more modern channels alone may not reach all intended audiences. Strategies to best reach vulnerable groups should be carefully considered when communicating to the public during and after a disaster [29].

Importantly, access to certain communications channels can be disrupted during disasters, and may not be restored before mold starts to grow; molds will grow on materials or surfaces that remain moist for at least 24–48 h [12, 32]. Although these FGDs did not assess feedback on the use and efficacy of text message communication, previous research shows that text messaging can be essential to early disaster communications, though it may not always be effective at reaching vulnerable populations [33]. Altogether, dissemination of messages through various methods, including both traditional (e.g., radio and television) and more contemporary (e.g., social media and the Internet), might prove beneficial in reaching a diverse range of audiences. Communication materials should also use plain language that specifically targets and appeals to the intended audiences to help increase the likelihood of recommended mold remediation practices.

These FGDs suggested that there may be opportunity to expand upon existing public health messaging about how to protect oneself from mold exposure, particularly for people who experience enduring mold growth in their homes and are unable to leave. Although current federal communication materials offer guidance for practicing safe mold clean-up following a natural disaster, these materials are intended to be used as emergency response tools in the immediate aftermath of a flooding event [12, 34, 35]. Thus, the information included in these materials was not relevant at the time of the FGDs, as participants had been dealing with long-term mold growth for several years. Some participants noted that existing materials are written for readers who have some degree of choice of whether or not to live in a home with mold, whereas many participants were living in homes with mold and were constantly exposed. Additionally, not all existing materials denote their purpose as disaster response tools. Federal guidance for persons who are unable to leave their homes during mold remediation or are unable to remediate mold might prove beneficial.

This analysis is subject to several limitations. Results of these FGDs reflected individual beliefs and are therefore not generalizable to other populations outside of this analysis. Data about the extent of flooding in participants’ residences were not collected, and individual experiences with post-hurricane mold clean-up may have varied substantially. FGDs took place nearly three years after Hurricane Harvey landfall, and comments about the immediate aftermath of the flooding event and short-term mold remediation may therefore be subject to recall bias. Given the particular risk for IMIs among immunocompromised people, future research to address this population’s perceptions about mold, mold clean-up, and federal communications materials would be beneficial.

## Conclusions

Overall, these FGD results suggest a need for wider, strategic, and timely dissemination of public health materials about mold and recommended clean-up practices following a natural disaster. Relevant public health messaging may influence modifying factors and perceptions related to mold’s impact on health and clean-up practices and improve adherence to remediation guidance. Expanded federal communication about the risks of ongoing mold exposure is also needed. Preparedness and prevention messaging should be disseminated before and during hurricanes and flooding events, and messaging about mold clean-up and long-term mold exposure should be disseminated following events. Additionally, FGD results highlighted older populations’ preference for more traditional methods of communication (e.g., radio, in-person) following Hurricane Harvey. Targeted messaging and outreach through these types of communication channels might allow older populations to more easily access assistance and guidance for participating in proper and safe mold clean-up following disaster events. However, given the limited reach and resource-intensive nature of some traditional communication methods, it is also important to maintain readily available written materials that can be accessed at any time on a website and disseminated in print in preparation for a natural disaster. Similar messaging strategies about how to access financial assistance may also be useful to reduce instrumental barriers to mold clean-up. Further research is needed to better understand the public’s perceptions about mold and its health effects. Increased awareness about mold-related illnesses may result in reduced delays in diagnosis and better patient outcomes.

## Data Availability

The dataset used and analyzed during the current study is available from the corresponding author on reasonable request.

## Abbreviations

IMI: Invasive mold infection
CDC: Centers for Disease Control and Prevention
SES: Socioeconomic status
PPE: Personal protective equipment
FGD: Focus group discussion
EPA: Environmental Protection Agency
HHD: Houston Health Department
HBM: Health Belief Model
EPPM: Extended Parallel Process Model
PPM: PRECEDE-PROCEED Model
